# Potential Chronotherapeutic Optimization of Antimalarials in Systemic Lupus Erythematosus: Is Toll-Like Receptor 9 Expression Dependent on the Circadian Cycle in Humans?

**DOI:** 10.3389/fimmu.2018.01497

**Published:** 2018-07-06

**Authors:** Erika Aurora Martínez-García, Maria Guadalupe Zavala-Cerna, Andrea Verónica Lujano-Benítez, Pedro Ernesto Sánchez-Hernández, Beatriz Teresita Martín-Márquez, Flavio Sandoval-García, Mónica Vázquez-Del Mercado

**Affiliations:** ^1^Instituto de Investigación en Reumatología y del Sistema Músculo Esquelético, Centro Universitario de Ciencias de la Salud, Universidad de Guadalajara, Guadalajara, Mexico; ^2^Departamento de Fisiología, Centro Universitario de Ciencias de la Salud, Universidad de Guadalajara, Guadalajara, Mexico; ^3^UDG-CA-703, Inmunología y Reumatología, Centro Universitario de Ciencias de la Salud, Universidad de Guadalajara, Guadalajara, Mexico; ^4^Immunology Research Laboratory, Programa Internacional de Medicina, Universidad Autonoma de Guadalajara, Guadalajara, Mexico; ^5^Laboratorio de Inmunología, Centro Universitario de Ciencias de la Salud, Universidad de Guadalajara, Guadalajara, Mexico; ^6^Departamento de Clínicas Médicas, Centro Universitario de Ciencias de la Salud, Universidad de Guadalajara, Guadalajara, Mexico; ^7^UDG CA-701, Inmunometabolismo en Enfermedades Emergentes (GIIEE), Centro Universitario de Ciencias de la Salud, Universidad de Guadalajara, Guadalajara, Mexico; ^8^Hospital Civil de Guadalajara “Juan I. Menchaca”, Servicio de Reumatología, Programa Nacional de Posgrados de Calidad (PNPC), Consejo Nacional de Ciencia y Tecnología (CONACYT), Guadalajara, Mexico

**Keywords:** toll-like receptor 9, systemic lupus erythematosus, circadian cycle, chronotherapy, chloroquine

## Abstract

Toll-like receptor 9 (TLR9) belongs to the group of endosomal receptors of the innate immune system with the ability to recognize hypomethylated CpG sequences from DNA. There is scarce information about TLR9 expression and its association with the circadian cycle (CC). Different patterns of TLR9 expression are regulated by the CC in mice, with an elevated expression at Zeitgeber time 19 (1:00 a.m.); nevertheless, we still need to corroborate this in humans. In systemic lupus erythematosus (SLE), the inhibitory effect of chloroquine (CQ) on TLR9 is limited. TLR9 activation has been associated with the presence of some autoantibodies: anti-Sm/RNP, anti-histone, anti-Ro, anti-La, and anti-double-stranded DNA. Treatment with CQ for SLE has been proven to be useful, in part by interfering with HLA-antigen coupling and with TLR9 ligand recognition. Studies have shown that TLR9 inhibitors such as antimalarial drugs are able to mask TLR9-binding sites on nucleic acids. The data presented here provide the basic information that could be useful for other clinical researchers to design studies that will have an impact in achieving a chronotherapeutic effect by defining the ideal time for CQ administration in SLE patients, consequently reducing the pathological effects that follow the activation of TLR9.

## Introduction

The main role of the immune system is to identify and eliminate health threats through mechanisms of both adaptive and innate immunity (1, 2). The adaptive immune system specifically recognizes pathogens through T cell receptors and B cell receptors, while for the innate immune system, the use of pattern-recognition receptors (PRRs) has long been identified to help recognize pathogen-associated molecular patterns (PAMPs) and damage-associated molecular patterns (3). Toll-like receptor 9 (TLR9) is a PRR that recognizes hypomethylated CpG-DNA sequences in bacteria, viruses, and host DNA, which favor TLR9 signaling when they are included in immune complexes (4–7). Moreover, it has been proposed that TLR9 might be responsible for the initiation of autoimmunity, particularly in systemic lupus erythematosus (SLE), where the production of autoantibodies against double-stranded DNA (dsDNA) is a common characteristic (8). Historically, the use of antimalarial drugs (AMDs) such as chloroquine (CQ) and its analogs has been shown to be effective in the treatment of autoimmune diseases such as SLE (9–11). In general, it has been suggested that CQ could inhibit the endosomal acidification that is necessary for intracellular antigen processing and presentation (12). However, for the inhibition of TLR9 activation, acidification might not be the most important factor, since TLR9 requires contact with its ligand, and CQ has been shown to interfere by masking the TLR9-binding sites on the ligands. Therefore, this pathway has been described as one of the mechanisms through which CQ decreases the inflammatory response (13). It has been widely reported that cells and proteins of the immune system are regulated by the circadian cycle (CC) (14, 15); however, there are few studies that describe the impact of TLR9 circadian regulation and the therapeutic repercussions for SLE. This perspective deals with the evidence of TLR9 expression patterns related to CC and the interference of CQ in TLR9 activation, suggesting that in theory, it is possible to improve the benefit of CQ treatment based on its chronotherapeutic effect, and this might be exploited to reduce the activation of TLR9 that includes the production of autoantibodies and inflammatory cytokines in SLE. This information will be useful to conduct future clinical studies to achieve the best treatment results with CQ.

## Toll-Like Receptor 9

Toll-like receptor 9 identification occurred during homology structure studies on different Toll-Like Receptors (TLRs) (4, 16–18). TLRs are highly conserved proteins by means of positive selection induced *via* gene duplication (19–22). The human *TLR9* (*hTLR9*) gene is localized on chromosome 3p21.3 and consists of two exons that encode for 1,032 amino acids (aa) (18). There are five reported isoforms of TLR9, produced by alternative splicing: TLR9A to TLR9E, with variable expression in B and T cells (18, 23, 24). The protein structure of TLR9 has three domains: (1) an extracellular domain with leucine-rich repeats that recognizes pathogens, (2) a single transmembrane domain, and (3) an intracellular toll-interleukin 1 receptor (TIR) domain for signal transduction (25).

**Figure 1:**
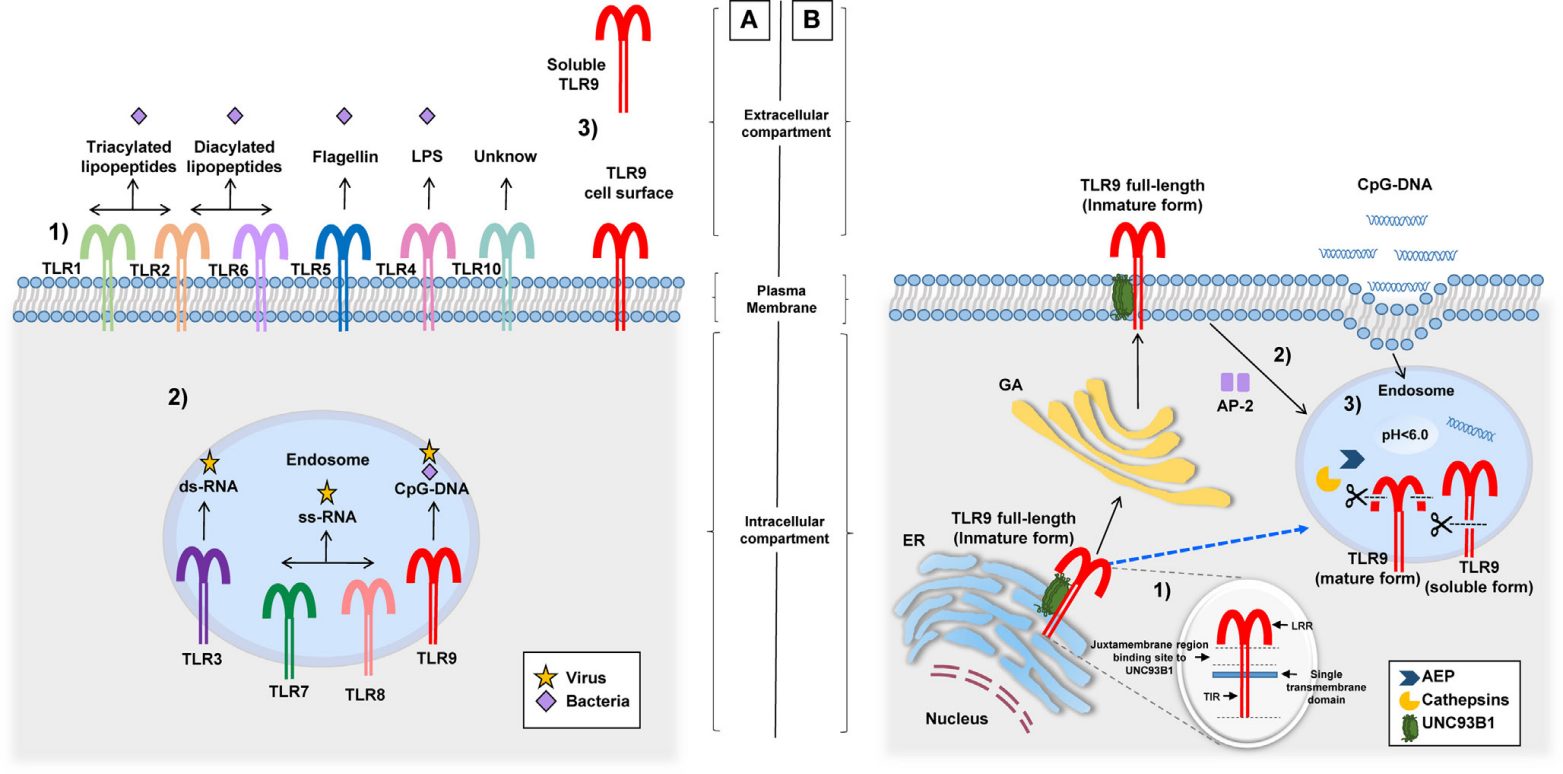
Different locations of TLRs and the TLR9 signaling route. **(A)** (1) TLRs (and their respective ligand) expressed on the cell surface: TLR1/TLR2 (triacylated lipopeptides), TLR2/TLR6 (diacylated lipopeptides), TLR5 (flagellin), TLR4 (LPS), and TLR10 (unknown). (2) TLRs expressed in endosomes: TLR3 (ds-RNA), TLR7/TLR8 (ss-RNA), and TLR9 (CpG-DNA). (3) TLR9 cell surface expression related to their signaling route and soluble form identified in pleural effusions and HEK 293 cells, although its production and functions are unknown. **(B)** Before TLR9 is located inside the endosome, it can be identified in the ER, the GA, and lysosomes. (1) Inside the ER, the juxtamembrane region of TLR9 interacts with UNC93B1, a facilitator protein that allows the exit of TLR9 toward the cell surface or directly to endosomes (dotted blue line). (2) Once on the cell surface, TLR9 becomes associated with AP-2 for cell internalization through the interaction with phagosomes or endosomes. (3) In this location, TLR9 modifies its structure by the action of AEP and cathepsins generating a mature form (80 kDa) and a soluble form (100 kDa) without transmembrane and TIR domains, this form is retained in the endosome and prevents the activation of TLR9. Abbreviations: ER, endoplasmic reticulum; GA, Golgi apparatus; LPS, lipopolysaccharide; HEK 293, human embryonic kidney 293; UNC93B1, unc-93 homolog B1; AP-2, adaptor protein complex 2; AEP, asparagine endopeptidase; TLR9, toll-like receptor 9; TIR, toll-interleukin 1 receptor; LRR, Leucine-rich repeats.

According to various studies, hTLR9 expression is predominant in the spleen, lymph nodes, tonsil, skin (keratinocytes), kidney, and peripheral blood leukocytes [dendritic cells (DCs), B cells, macrophages, neutrophils, eosinophils, natural killer, and T cells] (26–34). In all of them, TLR9 elicits their activation after engaging PAMPs. Despite the typical intracellular localization of TLR9 in endosomes, two additional sites of expression were described: (1) the cell surface of human and mouse neutrophils, intestinal epithelial cells (IEC), mouse colonic tissue, and hepatocellular carcinoma cells (35–37) and (2) a soluble form in bacterial pleural effusions and human embryonic kidney 293 cells (Figure 1A) (38, 39). The different localizations of TLR9 possibly have a role in its function (40), a concept that will be discussed in detail in the following section.

## TLR9: The Path to Endosomes for Ligand Identification and Activation

Endosomes or phagosomes are cell structures that contain PAMPs derived from phagocytosed pathogens. Inside these structures is where TLR9 engages targets and elicits cell activation (41, 42). Importantly, in studies performed with laser scanning confocal microscopy, it was found that before TLR9 interacts with endosomes or lysosomes, it is located in its full-length form (immature) inside the endoplasmic reticulum and/or the Golgi apparatus (GA) (43, 44) (Figure 1B). TLR9 is then translocated with unc-93 homolog B1 (UNC93B1), a facilitator protein, from the GA toward the cell surface, where it is associated with an adaptor protein complex 2 for internalization to endosomes (45–47). The binding between TLR9 and UNC93B1 depends on the presence of a specific sequence of aa located in the juxtamembrane region of TLR9. This finding became evident after the observation that mutated proteins with changes in the aa residues from Asp^812^ to Ser (D812S) and from Glu^813^ to Thr (E813T) in mice avoided the interaction between TLR9 and UNC93B1 and disrupted the continuous trafficking of TLR9 toward the endosome (48). There is still missing information as to whether this intracellular trafficking is required for other TLRs since, in the case of TLR7, for example, although it interacts with UNC93B1 protein, transportation from the GA to endosomes occurs without cell surface expression (46, 49). Once TLR9 is located in endosomes, it undergoes proteolytic cleavage performed by cathepsins (50, 51), generating a protein of 80 kDa, which is required for the adequate functioning of the receptor (52). Other proteolytic cleavage sites by cathepsins have been described in the aminoterminal region (NH_3_1-723 aa) of TLR9, producing a protein of 100 kDa that lacks the transmembrane and TIR domains, i.e., a soluble form retained in endosomes that inhibits TLR9 signaling (39). Importantly, the proteolytic function of cathepsins and other enzymes involved in the activation and signaling of TLR9 within the endosome microenvironment are carried out at an acidic pH (5.0 ± 0.2) (50, 53, 54). It is clear that the endosomal pH is an important factor for the activation of TLR9; however, when referring to TLR9 being expressed on the cell surface, there are no conclusive studies on its function or form of activation. However, one study performed in human hepatocellular carcinoma cell lines concluded that the expression of TLR9 on hepatocyte cell surface promotes tumorigenesis and cancer progression by promoting cellular proliferation and cell survival after receptor stimulation with CpG-oligodeoxynucleotides (CpG-ODNs) (55). Other studies suggest that cell surface expression of TLR9 is a rescue mechanism for the activation of neutrophils when their ligands are not internalized to endosomes or when intracellular TLR9 shows resistance to activation (35). Once endosomal TLR9 is sensitized by its ligand, it triggers MyD88-dependent signaling that induces the production of pro-inflammatory cytokines after activation of NF-kB (56–59).

## SLE, AMDs, and the Inhibition of TLR9

Loss of immunological tolerance in SLE is responsible for the secretion of circulating autoantibodies against cellular components such as nucleosomes, histones, ribonucleoproteins, DNA, and RNA helicase A, among others (60–63). One of the theories for autoantibody generation in SLE is the inefficient removal of cellular debris after apoptosis and neutrophil extracellular traps, two different processes in origin that could lead to an increased amount of free DNA and RNA when there is a defective removal of debris by macrophages (64–69). The principal autoantibodies associated with impaired clearance of cellular antigens in SLE are Sm/RNP, histone, Ro/La, and dsDNA (70–72). Artificial autoantibody production against nuclear antigens was described with the use of hydralazine. The mechanism proposed was the inhibition of the ERK signaling pathway with hydralazine, which caused a downregulation of the DNA methyltransferase 1 (*DNMT1*) mRNA, necessary for DNA methylation (73–75). It is important to acknowledge that hypomethylated DNA is a PAMP recognized by TLR9. In addition, both in humans and mice, TLR9 is involved in inflammation *via* the synthesis of inflammatory cytokines and activation of autoreactive B cells, contributing to autoantibody production and the subsequent clinical development of autoimmune features (76). In murine models of lupus-prone and mixed bone marrow chimeras, it became evident that when there was a lack of expression of endosomal TLRs, autoantibody production was absent. In the absence of TLR7, mice failed to generate anti-Sm/RNP autoantibodies and mice lacking TLR9 failed to produce anti-dsDNA autoantibodies (77, 78). The mRNA expression of *TLR7* and *TLR9* in SLE patients was associated with testing positive for anti-extractable nuclear antigens and anti-dsDNA, respectively (79). In addition, in kidney biopsies from patients with lupus nephritis (LN), there was evidence of TLR3, TLR7, and TLR9 overexpression with a positive correlation between TLR9 expression and high activity, measured by renal-systemic lupus erythematosus (R-SLEDAI) (80). Furthermore, SNPs in the *TLR9* gene, such as rs352140, were associated with LN (81).

In recent studies performed on peripheral blood mononuclear cells (PBMCs) from SLE patients and healthy individuals, there was evidence of elevated expression of TLR9 protein and mRNA, with a positive correlation to antinuclear antibodies titers (82–84). Therefore, previous studies have proposed that the modulation or inhibition of TLR9 is a potential tool for SLE treatment (85, 86). CQ was introduced as one of the AMDs that later proved to be beneficial for rheumatic diseases, mainly owing to an anti-inflammatory, immunosuppressive, and skin photoprotective effect (9, 87, 88). The administration of AMDs in SLE patients is indicated when there are no major organ manifestations (89), without standardized time for the prescription of this drug. The lipophilic non-protonated form of CQ is diffused in a passive way to endosomes, lysosomes, or Golgi vesicles, where it is protonated and retained by ion trapping (90, 91), suggesting that this protonated form of CQ could change the acidic medium necessary for the proteolytic processing of TLR9 in endosomes. However, the cleavage of TLR9 is not inhibited by AMDs. Studies have shown that TLR9 inhibitors such as CQ are able to mask TLR9-binding sites on nucleic acids (13). It is important to explore whether CQ, in addition to the findings mentioned above, has other mechanisms related to the inhibition of TLR9. In this respect, in a mouse model of sepsis, CQ administration induced decreased expression of TLR9 in the spleen, which was associated with increased survival and reduction of renal injury; interestingly, the same effect was evident in the absence of TLR9 (TLR9-deficient mice) (92). Nonetheless, this effect has not been acknowledged in autoimmune diseases or other immune system-related pathologies.

## TLR9 Circadian Behavior and Synchronization with CQ Administration

The CC is known to regulate the main biological processes (physiological, metabolic, and behavioral) in living organisms, originated by oscillations of light and dark conditions within a 24-h period, which is aligned with the rotation of the earth on its own axis (93–95). Recently, the importance of the CC has been highlighted by the recipients of the Nobel Prize in Physiology and Medicine 2017: Jeffrey Hall, Michael Rosbash, and Michael Young. These investigators stated that the CC is a molecular genetic mechanism that directs various functions in *Drosophila melanogaster* (96–100), and knowledge of this has had an impact on the clinical course and treatment of the human diseases. The master regulator of the CC is localized in the suprachiasmatic nucleus (SCN) of the hypothalamus and the activity of this regulator depends on the received light through photoreceptors in the retina of both eyes (101–104). The SCN is also synchronized with other peripheral circadian clocks such as the hypothalamic–pituitary–adrenal axis and the immune system (105, 106). The molecular mechanisms of these peripheral circadian clocks are autonomous and controlled by a transcriptional–translation feedback loop in clock genes (107, 108). Some of these clock genes include the heterodimer circadian locomotor output cycles kaput gene and brain and muscle aryl hydrocarbon receptor nuclear translocator 1 gene (*Clock*:*Bmal1)* (109–111). This heterodimer functions as a transcriptional factor for the Period (*Per*) and Cryptochrome (*Cry*) genes (112, 113), whose proteins then become transcriptional repressors of the *Clock:Bmal1* heterodimer, allowing repeating of the cycle (114).

An experimental analysis performed in mouse tissues (aorta, adrenal gland, brainstem, fat, cerebellum, heart, hypothalamus, kidney, liver, lung, and skeletal muscle) demonstrated that the patterns of gene expression in 43% of the entire mouse genome had a circadian behavior (115). Particularly when referring to the immune system and the CC, it was observed that the number and functions of leukocytes are controlled by clock genes; therefore, they are subjects of this CC (116). Furthermore, after analyzing mouse peritoneal macrophages by microarray, 8.1% of expressed genes in these cells had circadian control, including *Bmal1, Clock, Per1, Per2, Cry1*, and *Cry2*, among others (117). There is evidence that the expression of these clock genes in mouse macrophages, DCs, and B cells is observed at two peaks every 12-h under light–dark conditions (118). More specifically, the percentage of neutrophils and their phagocytic function increase after dark conditions, a phenomenon that occurs in a cyclic manner (119).

Silver et al. in 2012 published their results describing that peritoneal macrophages derived from *Per2*-mutant mice (*mPer2^Brdm1^*) that were subject to conditions of 12-h light/12-h darkness and challenged with CpG-ODNs (TLR9 ligand) at different times, had a fluctuation in the expression of *TLR9* mRNA, with peaks at Zeitgeber time (ZT) 11 (5:00 p.m.), which correlated with the production of low cytokine levels (TNF-α and IL-12); yet, this circadian behavior was not observed in TLR1, 2, 3, 4, 5, 6, and 7 (120). In addition, they reported an increase in the expression of *TLR9* mRNA and median fluorescence intensity at ZT19 (1:00 a.m.) in spleen cells compared with that at ZT7 (1:00 p.m.) (120). Moreover, the mRNA expression of *TLR1*–*TLR5* and *TLR9* (not *TLR6* and *TLR7*) in IEC presents a circadian pattern with higher levels at ZT0 (6:00 a.m.) vs. ZT12 (6:00 p.m.) and is dependent on the presence of retinoic acid receptor-related orphan receptor-α (121). There is a gap in the knowledge of circadian TLR9 expression. We consider that it would be interesting to research this concept in mice in depth. However, the studies performed so far have not been examined in the context of human SLE, either in the lupus-prone NZBxNZW or pristane-induced lupus models, to evaluate the role of circadian expression of TLR9 in autoimmunity.

All these results on TLR9 expression confirm the existence of circadian behavior in other organs, independent of the SCN action. A possible candidate for other anatomical regions capable of capturing the external light that entails circadian pathways in humans is the skin (122–124). In a scenario of autoimmunity, such as that in SLE, injury to the skin is the second most frequent clinical feature, which might even induce lupus flares (125). One of the main environmental factors associated with a lupus flare is UV light exposure (126), which causes DNA damage and subsequent apoptosis of keratinocytes (sunburn cells), being a source of nuclear autoantigens that undergo relocalization of autoantigens such as Ro (125–128). In this respect, *in vivo* studies showed that erythema induced after fixed doses of UV light exposure caused an exacerbated inflammatory response in the morning in comparison with that in the afternoon (129), suggesting that the skin inflammation processes depends on a CC and not entirely on UV light exposure. Other autoimmune rheumatic diseases associated with environmental triggering factors are the idiopathic inflammatory myopathies that are more prevalent near equatorial zones (130) since UV light exposure seems to be one important factor associated with its clinical presentation. Indeed, our group has reported the high prevalence of anti-Mi-2 antibodies in these subsets of autoimmune rheumatic diseases (131).

On the other hand, it is well known that the CC influences the pharmacodynamics of drugs (132, 133), which gives rise to the term chronotherapy, defined as the administration of drugs according to biological clocks: daily, monthly, seasonal, or yearly, leading to the maximum benefit and reduction of adverse effects (134). Perhaps the best clinical example is the optimization of glucocorticoid (GC) doses in patients with autoimmune conditions, including SLE, where the production of cortisol by the host allows a decrease in GC demands, therefore, providing a chronotherapeutic effect (101, 133, 135, 136). In malaria patients, the influence of CQ in the development of the parasite in erythrocytes, manifestations of the disease, and the timing effect of the drug were demonstrated in 1991, highlighting its chronotherapeutic effect for the elimination of the parasite (137).

## Conclusion and Perspectives

**Figure 2:**
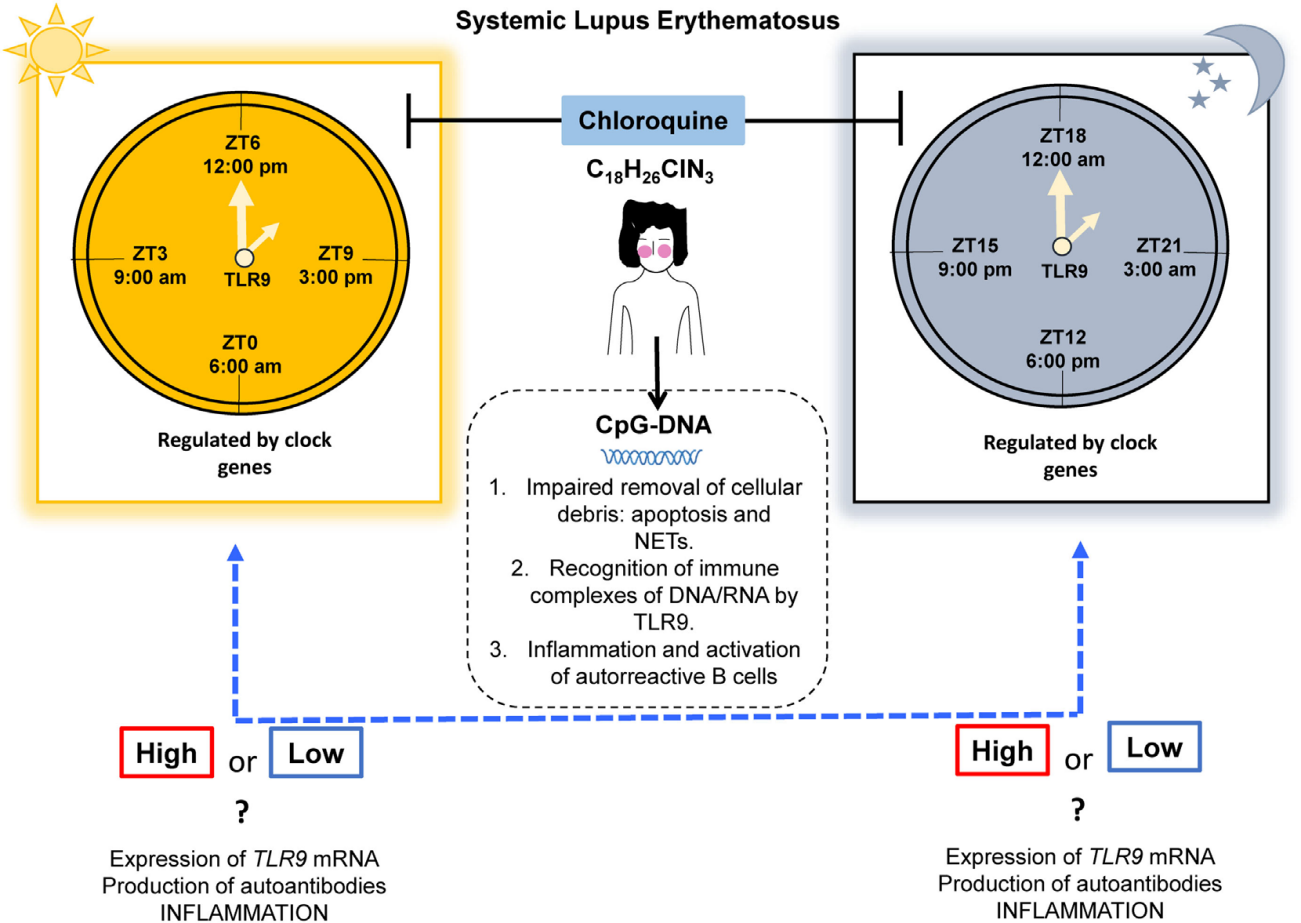
Proposal for a chronotherapeutic effect in SLE patients receiving CQ treatment. Ligands for TLR9 include CpG-DNA, which might originate during impaired clearance of cellular debris. The expression of TLR9 has shown circadian behavior in mice, with higher expression at ZT19 (1:00 a.m.) and lower expression at ZT7 (1:00 p.m.) and this is regulated by clock genes. However, in humans, this information is lacking. CQ acts as an inhibitor of TLR9 by masking its binding sites for nucleic acids. We propose that the chronotherapeutic optimization of CQ would make it more beneficial if it is prescribed when there is maximal expression of TLR9 in SLE that possibly leads to a reduction in the expression of TLR9, autoantibodies production, and inflammation (dotted blue line). Abbreviations: CQ, chloroquine; SLE, systemic lupus erythematosus; ZT, Zeitgeber time; NETs, neutrophil extracellular traps; TLR9, toll-like receptor 9.

It would be extremely interesting to verify whether time-restricted expression of TLR9 and clock gene regulation is present in human beings. These findings could be demonstrated with clock-adjusted gene expression analysis in PBMCs, which might support this chronotherapeutic regimen in SLE, by increasing the modulating effect of autoantibody production and inflammation (Figure 2). Also, we propose to study the TLR9 expression and signaling pathways in non-lupus prone mouse strains such as the pristane-induced murine lupus model, owing to its feasibility. Here, we provided the basic evidence that different expression patterns of TLR9 are sustained in association with the CC in mice. Nevertheless, this finding still needs to be addressed in humans, taking into account the following key points: (1) the expression of TLR9 is controlled by the CC, (2) inflammatory cytokine production correlates with the expression of TLR9, (3) CQ has an anti-inflammatory effect by disrupting the signaling of intracellular TLR9, and (4) CQ is already used as monotherapy or combination therapy for autoimmune diseases. However, there is no consensus with respect to the ideal time for AMDs prescription.

## Ethics Statement

All cited studies reported in the present perspective where conducted in compliance with relevant Ethical Guidelines. This article does not represent work made by the authors with human or animal subjects.

## Author Contributions

Conceived and designed the idea: EM-G, MZ-C, AL-B, PS-H, BM-M, FS-G, and MV-DM. Conducted the bibliographic search: EM-G and AL-B. Analysis and discussion of the information: EM-G, MZ-C, AL-B, PS-H, BM-M, and MV-DM. Wrote the paper: EM-G, MZ-C, AL-B, and MV-DM. Figure editing EM-G, AL-B, and FS-G. All the authors declare that they have read and approved the final version of the manuscript and that they are responsible for its content.

## Conflict of Interest Statement

The authors report no conflicts of interest. The authors alone are responsible for the content and writing of the paper.
